# Rapid Eye Movement Sleep Deprivation Combined With Fluoxetine Protects Against Depression-Induced Damage and Apoptosis in Rat Hippocampi *via* A1 Adenosine Receptor

**DOI:** 10.3389/fpsyt.2021.599399

**Published:** 2021-07-16

**Authors:** Xuan Ju, Shengdong Wang, Pan Yan, Chunyan Zhu, Xiwen Hu, Jiezheng Dong, Zhonglin Tan

**Affiliations:** ^1^Psychiatric Department, Hangzhou Seventh People's Hospital, Mental Health Center of Zhejiang University School of Medicine, Hangzhou, China; ^2^Molecular Biology Laboratory, Hangzhou Seventh People's Hospital, Mental Health Center of Zhejiang University School of Medicine, Hangzhou, China

**Keywords:** depression, sleep deprivation, fluoxetine, hippocampus, A1 adenosine receptor

## Abstract

**Background:** Rapid eye movement sleep deprivation (REMSD) and fluoxetine affect depression, yet the detailed molecular mechanisms were not clear.

**Methods:** Rat depression chronic unpredictable stress was constructed, and the body weight of rats was measured. The efficacy of REMSD and fluoxetine on the pleasure experience, exploration, and cognition of rats with depression was determined by the Sucrose preference test, the open field test, and Morris water task, respectively. The effects of REMSD and fluoxetine on depression-induced damage and apoptosis in rat hippocampi were detected using hematoxylin–eosin staining and terminal transferase-mediated biotin 2′-deoxyuridine, 5′-triphosphate nick end labeling. A1 adenosine receptor content was measured by immunohistochemistry. Relative expressions of the A1 adenosine receptor, proteins related to apoptosis (B Bcl-2-associated X protein; B-cell lymphoma 2), phosphoinositide 3-kinase, P38 mitogen-activated protein kinase, cFos, and adenosine deaminase RNA specific two were quantified by quantitative real-time polymerase chain reaction and Western blot as needed.

**Results:** Depression decreased rat weight. REMSD combined with fluoxetine increased body weight, prompted rat behavior, alleviated depression-induced damage, attenuated apoptosis, and promoted A1 adenosine receptor level in rat hippocampi. Furthermore, the combined therapy upregulated expressions of A1 adenosine receptor, B-cell lymphoma 2, and phosphoinositide 3-kinase but downregulated those of B-cell lymphoma 2-associated X protein, P38 mitogen-activated protein kinase, cFos, and adenosine deaminase RNA specific 2 in the hippocampi of rats with depression.

**Conclusion:**REMSD combined with fluoxetine protected rats against depression-induced damage and apoptosis in the hippocampus *via* the A1 adenosine receptor, providing a possible treatment strategy for depression.

## Introduction

Depression has been recognized as one of the most significant causes of emotional suffering and a factor affecting the morbidity of several medical disorders (1, 2). Patients diagnosed with depression have been shown to lose interest in physical activities, lose appetite or overeat, have troubles with concentration, memory, and decision-making, and even have a tendency to commit suicide (3). Also, recent years have witnessed increases in both the number and geographic range of the population who suffer from depression; thus, it is of great urgency and significance to develop a therapeutic method for preventing and treating depression (4).

Various researches on epidemiology unveiled the relation of sleep duration with health-related conditions, such as cardiovascular health, mental disorders, and mortality (5–7). Also, previous studies have shown the association between sleep and depression, providing evidence for the roles of sleep in the development and progression of depression (8). Total sleep deprivation (TSD) is the complete disruption of one or both types of sleep: rapid eye movement (REM) sleep and non-REM sleep. At present, TSD is regarded as the most widely documented rapid-onset antidepression therapy (9). However, cumulative TSD could result in an increased risk of major depression (10). As suggested previously, the combination therapy of TSD and other therapy, light therapy, for instance, have positive short-term and long-term effects even in patients who have been diagnosed with treatment-resistant depression (11). Meanwhile, antidepressants have been acknowledged and used as the standard treatment for major depressive disorders (MDDs); however, their efficacy tends to be variable and incomplete (12). As one of the most prescribed antidepressants, fluoxetine plays a vital role in MDD treatment and has been approved for clinical use, with effects on neurocognitive functions in adults with depression (13, 14). Additionally, the combined therapy of TSD and fluoxetine has been used for the treatment of depression (15). REM sleep changes have been considered as true endophenotypes of depression (16). Shortened REM latency, elevated REM sleep duration, and increased REM density have been regarded as biological markers of depression that might predict relapse and recurrence (16). Thus, the combined therapy of REM sleep deprivation (REMSD) and fluoxetine used for the treatment of depression and the potential mechanism has been needed to be explored.

Adenosine, an endogenous autacoid, can affect almost all aspects of cellular physiology, and its effects can be triggered *via* enrolling the four G protein-coupled receptors (A1, A2A, A2B, and A3) (17). A1 adenosine receptor has been found to mediate various physiological processes, allowing it to be a promising target of drugs for different pathological conditions (18). Several pieces of evidence suggested that the therapeutic effects of different non-pharmacological treatments of depression, such as TSD and electroconvulsive therapy, are modulated by A1 adenosine receptor upregulation or activation (19). Meanwhile, the A1 adenosine receptor, along with TSD and other medications, has a suppressive effect on depression-like behavior in mice (20). However, the interaction between the combination therapy of REMSD and fluoxetine and the A1 adenosine receptor remained to be further addressed. In our current study, we constructed a rat with depression by chronic unpredictable stress (CUS) and then treated rats with REMSD and fluoxetine alone or together, hoping to discover the efficacy of the combination therapy of REMSD and fluoxetine on rats with depression and develop a potential therapeutic method for depression in clinical practice.

## Materials and Methods

### Ethics Statement

All the animal experiments were performed in line with the guidelines of the China Council on Animal Care and Use. The Committee of Experimental Animals of Zhejiang Chinese Medical University has approved our current study (JSK2019091001). Every effort was made to minimize pain and discomfort of the animals. All animal experiments were performed at Zhejiang Chinese Medical University.

### Animal Model Construction

In the current study, male Sprague-Dawley rats (3 months, 310–420 g, *n* = 50) were purchased from the Experimental Animal Center of Henan Province [license number: SXCK (Henan) 2012–0011] and housed in pathogen-free cages (two/cages) under the conditions as follows: 12-h day/light cycle, 22–26°C, and 50–60% humidity. Before our study, all rats were allowed to have free access to a standard experimental diet and sterile tap water.

For the subsequent study, fluoxetine (F132) was obtained from Sigma-Aldrich (St. Louis, MO, USA), and the rats were randomly assigned to five groups (*n* = 10 for each group): control, CUS, CUS + REMSD, CUS + fluoxetine, and CUS + REMSD + fluoxetine groups. Rats in the control group were housed normally without any treatment, and rats in the remaining groups were subjected to depression model construction as described in a prior study (21). For depression induction, the rats were exposed to a random type of CUS for 28 days. Types of CUS in this study were as follows: (a) behavioral restriction for 2 h; (b) foot electrocution (current intensity: 1.0 mA, frequency: 1 s each time, six times per minute, 10 min in total); (c) cage tilting (45° relative to the vertical axis) for 24 h; (d) food deprivation for 24 h; (e) water deprivation for 24 h and empty water bottle exposure for 1 h after a period of acute water deprivation; (f) forced swimming in ice water (water temperature was 4°C) for 5 min; (g) wet bedding (with 300 ml of water spilled in the bedding) for 10 h (night cycle); (h) reverse of light/dark cycle for 24 h; (i) horizontal shake of cage (one time per second for 15 min, with the temperature inside the cage set at 40°C); (j) tail clamping at 1 cm for 1 min. The body weight of the rats in the control and CUS groups was also recorded on days 1, 7, 14, 21, and 28 after model construction.

Then, rats in the CUS + REMSD and CUS + REMSD + fluoxetine groups were deprived of sleep using the REMSD method as previously described (22). A bucket (1.2 × 0.5 × 0.4 m) was used for REMSD, which contained 15 of the platform (diameter: 6.3 cm, height: 8.0 cm). In this phase, rats were kept individually on a small platform (diameter: 6.3 cm, height: 8.0 cm) surrounded by water (temperature: 22°C) in a basket and were given free access to food and water and allowed to move freely. For the control group, the small platforms were replaced with larger ones (diameter: 12.5 cm, height: 8.0 cm), and individual rats were kept on a bigger platform under identical conditions, allowing them to enter REM sleep without falling into the water. Due to the requirement of postural muscle atonia during REM sleep, rats were consequently unable to maintain an extended and relaxed body posture and tended to fall into the surrounding water. As a result, the rats woke up at the onset or before REM sleep, and therefore sleep deprivation was realized.

After 28 days of REMSD, the rats were allowed to sleep uninterruptedly in individual cages with sufficient water and food supply. Meanwhile, rats in the control, CUS, and CUS + REMSD groups were intraperitoneally injected with an equivalent volume of saline (07982, Sigma-Aldrich, USA), whereas rats in the remaining groups were intraperitoneally injected with fluoxetine (5 mg/kg/day) once a day for 28 days with or without REMSD, and their body weight was measured again on days 0 and 28 after the treatment with REMSD and fluoxetine.

Finally, all the rats were killed *via* inhalation of 5% isoflurane (792632, Sigma-Aldrich, USA), and hippocampi samples were collected and stored at 4°C after washing with phosphate-buffered saline (PBS, P5493, Sigma-Aldrich, USA). The experimental design is outlined in Supplementary Figure 1.

### Sucrose Preference Test

The sucrose preference test was performed to determine the capability of rats to experience pleasure in line with a prior study (23). In detail, on day 28 posttreatment with REMSD and fluoxetine, the rats were housed in individual cages during the dark cycle. A bottle containing 100 ml of 1% sucrose solution (S7903, Sigma-Aldrich, USA) was placed in each cage for 24 h, and the rats were allowed for adaptation. Then, the bottle was removed, and rats were deprived of food and water for 23 h. Then, two bottles, one contained 100 ml of 1% sucrose solution, whereas another contained 100 ml of tap water, and the bottles were placed in each cage for 1 h. The consumed sucrose solution and water were recorded, and the consumption of sucrose solution was calculated according to the formula: consumption = intake volume of sucrose solution (milliliter)/body weight (100 g).

### Open Field Test

A previous study showed that the open field test could be used to analyze the anxiety-like and locomotor behavior of the rats (24, 25). In our current study, the open field test was also conducted on day 28 after treatment with REMSD and fluoxetine to evaluate the exploration capability of rats. The open field, which consisted of a black lusterless box (100 × 100 × 60 cm), was divided into the central part (25%) and the rest of the box (75%). The open field test was conducted during the dark cycle, and a video camera, which was suspended ~200 cm above the field, was used for recording.

For the open field test, the acrylic box was cleaned using 5% ethanol (24194, Sigma-Aldrich, USA) before introducing rats, and then rats were placed at the corner of the open field facing the wall. The behavior of each rat was recorded for 5 min, and the data from the postrecording of the horizontal movement distance, immobility time (i.e., the time rats stay in the center of the open field), retention within 5 min, and numbers of rats entering the central region were analyzed. Immobility time represented motility, and higher scores of immobility time indicated severe depression.

### Morris Water Task

Morris water task was performed on day 28 post-REMSD, and fluoxetine treatment was used in accordance with previous research to test the cognition ability of rats (26). In our current study, the water maze consisted of a container (diameter: 1.6 m, height: 50 cm) filled with water (temperature: 21–22°C). The platform (diameter: 9 cm) was placed 1.5–2.0 cm below the water in the center of the container. The rats were given four trials per day, with a 30-s intertrial interval, for five consecutive days, and the escape latency (time to reach the platform) of each trial was measured. For the experiment, the rats were assigned to the Morris water task, in which rats were randomly placed into the pool facing the wall at one of four equidistant drop locations. Twenty-four hours after the hidden platform experiment, the platform was removed from the swimming pool. Rats were released on drop points on the opposite side of the platform quadrant. Rats were allowed to swim for a total of 90 s, and the number of times that the rat crossed the original platform in 90 s was recorded (space exploration).

### Hematoxylin–Eosin Staining

For hematoxylin–eosin (H&E) staining, hippocampus tissue of rats was harvested with caution, fixed in 4% paraformaldehyde (P6148, Sigma-Aldrich, USA), and finally embedded in pre-dissolved paraffin (1.07151, Supelco, Bellefonte, PA, USA). Samples were resected, stained by an H&E Stain Kit (C0105, Beyotime, Shanghai, China), and analyzed under an inverted light microscope (CKX53, Olympus, Tokyo, Japan) under × 100 and × 200 magnifications.

### Terminal Transferase-Mediated Biotin 2′-Deoxyuridine, 5′-Triphosphate Nick End Labeling Assay

Terminal transferase-mediated biotin 2′-deoxyuridine, 5′-triphosphate nick end labeling (TUNEL) assay was performed to evaluate apoptosis of cells with a TUNEL assay kit (ab206386, Abcam, Cambridge, UK) as previously described (27). The hippocampus tissue was first deparaffinized and treated with 20 μg/ml deoxyribonuclease-free proteinase K (AM2548, Thermo Fisher Scientific, Waltham, MA, USA) at room temperature for 20 min and was then washed with PBS three times (5 min for each). After being fixed at room temperature for 15 min, the samples were washed with PBS for another three times (5 min each time). Next, each sample was added with 50 μl of biotin labeling and incubated for 1 h at 37°C, followed by processing with a 3,3′-diaminobenzidine kit (DA1015, Solarbio, Beijing, China) and PBS washing. Then, the samples were sealed after clearing with methylbenzene (244511, Sigma-Aldrich, USA) for 5 min. The apoptotic cells were observed using a stereomicroscope (SZX10, Olympus, Japan) under × 100 and × 200 magnifications.

### Immunohistochemical Analysis

The collected hippocampus tissue was fixed in 4% paraformaldehyde solution overnight and then sliced into sections with a thickness of 5 μm, followed by embedding in paraffin for staining. The paraffin sections were stained with anti-A1 adenosine receptor antibody (ab124780, 1:1,000, Abcam, UK) at 4°C overnight and then stained using horseradish peroxidase-conjugated goat anti-rabbit secondary antibody (ab205718, 1:2,000, Abcam, UK) at room temperature and a 3,3′-diaminobenzidine kit for 15 min. The nuclei were counterstained using hematoxylin (H3136, Sigma-Aldrich, USA), and the positive cells in five randomly chosen fields were counted and observed with a stereomicroscope. Here, the sample size was *n* = 10.

### RNA Isolation and Quantitative Real-Time Polymerase Chain Reaction

Trizol (R0016, Beyotime, China) was used for extracting total RNA from hippocampus tissues based on the manuals of the manufacturer. The extracted RNA was preserved at −80°C, and the concentration was determined using a NanoDrop spectrometer (ND-LITE, Thermo Fisher Scientific, USA). Of total RNA, 1 μg was synthesized into complementary DNA using a complementary DNA synthesis kit (D7178S, Beyotime, China). Quantitative real-time polymerase chain reaction (QRT-PCR) was conducted using a BeyoFast^TM^ SYBR Green One-Step qRT-PCR kit (D7268S, Beyotime, China) in the AriaMx real-time PCR System (G8830A, Agilent Technologies, Santa Clara, CA, USA) under the following conditions: at 95°C for 10 min, and 40 cycles of at 95°C for 20 s and at 60°C for 30 s, with glyceraldehyde 3-phosphate dehydrogenase (GAPDH) as an internal reference. Primer sequences are listed in Table 1. Relative expressions were later calculated by the 2^−ΔΔCT^ method (28). Here, the sample size was *n* = 10.

**Table 1:** Primers for qRT-PCR.

**Gene**	**Primers (5**^**′**^**->3**^**′**^**)**
A1 adenosine receptor	
Forward	GTGATCAAGTGTGAGTTTGA
Reverse	AGAGGGTGATACAGTTCAAG
Bax	
Forward	TGAACAGATCATGAAGACAG
Reverse	ATGTTGTTGTCCAGTTCATC
Bcl-2	
Forward	GGTATGATAACCGGGAGAT
Reverse	CCAGTATCCCACTCGTAG
PI3K	
Forward	GTCTTCCTGAGAGCTTTAGA
Reverse	GATGCGTAGAATCTGTAAG
P38 MAPK	
Forward	GTATATACACTCGGCTGACA
Reverse	GGCATCCTGTTAATGAGATA
cFos	
Forward	CTCTAGTGCCAACTTTATCC
Reverse	GTAGGTGAAGACAAAGGAAG
ADAR2	
Forward	CACAGGTACAGATGTCAAAG
Reverse	ATGATGCTGGAGAAGTAGAT
GAPDH	
Forward	CTTGTGACAAAGTGGACAT
Reverse	TAGACTCCACGACATACTCA

### Western Blot

Protein expressions were measured by Western blot based on a previous study (29). After the collection of hippocampus tissue, the lysis and extraction for protein were performed using radioimmunoprecipitation assay lysis buffer (P0013C, Beyotime, China), and the concentration of protein was determined with a bicinchoninic acid protein kit (P0012S, Beyotime, China). Twenty micrograms of protein lysate samples were subsequently electrophoresed by sodium dodecyl sulfate-polyacrylamide gel electrophoresis (P1200, Solarbio, China) and transferred into a polyvinylidene fluoride membrane (YA1701, Solarbio, China). After blocking with fat-free milk (5%) for 2 h, the membrane was incubated with the following primary antibodies: anti-A1 adenosine receptor antibody (ab124780, 1:1,000, Abcam, UK), anti-Bcl-2-associated X protein (Bax) antibody Aria Mx (ab32503, 1:10,000, Abcam, UK), anti-B-cell lymphoma 2 (Bcl-2) antibody (ab182858, 1:2,000, Abcam, UK), anti-phosphoinositide 3-kinase (PI3K) antibody (ab151549, 1:1,000, Abcam, UK), anti-P38 mitogen-activated protein kinase (P38 MAPK) antibody (ab170099, 1:5,000, Abcam, UK), anti-cFos antibody (ab190289, 1:2,000, Abcam, UK), anti-adenosine deaminase RNA specific 2 (ADAR2) antibody (ab50610, 1:1,000, Abcam, UK), and anti-GAPDH antibody (ab181602, 1:10,000, Abcam, UK) at 4°C overnight, with GAPDH as an internal control. After that, the membrane was incubated with secondary horseradish peroxidase-conjugated antibodies: goat anti-rabbit immunoglobulin G H&L (1:1,000, A0208, Beyotime, China) and goat anti-rat immunoglobulin H&L (1:1,000, A0192, Beyotime, China) at room temperature for 1 h and subsequently washed using Tris-buffer saline Tween (ST673, Beyotime, China) for three times. After collecting the protein bands, an enhanced chemiluminescence kit (P0018FS, Beyotime, China) was used for visualization. The data were analyzed in the iBright CL1500 Imaging System (A44240, Thermo Fisher Scientific, USA), and gray values of the strips were further calculated using ImageJ (version 5.0, Bio-Rad, Hercules, CA, USA). Here, the sample size was *n* = 10.

### Statistical Analysis

The data were expressed as mean ± standard deviation (SD; *n* = 10). Statistics were analyzed using Graphpad prism 8.0 software (Graphpad Software, Inc., La Jolla, CA, USA). Comparison between two groups was performed using the Student's *t*-test. Comparisons between multiple groups were performed using a one-way analysis of variance, followed by a Dunnett's *post-hoc* test. The data were determined to be statistically significant when *P*-value < 0.05.

## Results

### Depression Decreased the Body Weight of Rats

**Figure 1:**
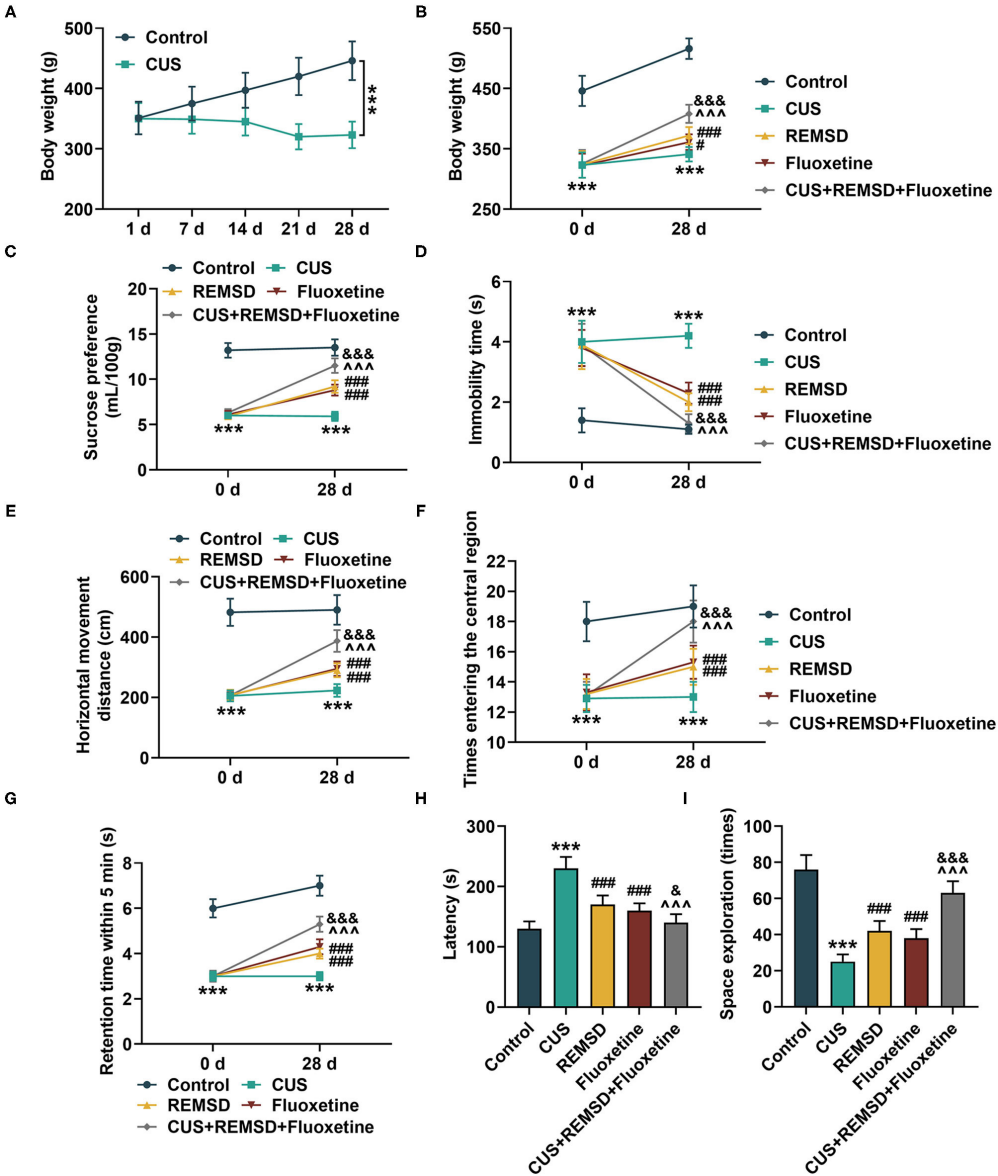
Depression reduced body weight in rats, and sleep deprivation combined with fluoxetine increased body weight, and prompted behavior of rats with depression. **(A)** Body weight of rats subjected to depression model construction or not was measured on days 1, 7, 14, 21, and 28 after depression model construction (*n* = 10 for each group). **(B)** Body weight of rats in control, CUS (depression), REMSD, fluoxetine, and REMSD + fluoxetine groups was measured. **(C–E)** Sucrose preference **(C)**, immobility time **(D)** and horizontal movement distance **(E)**, retention time in central area **(F)**, and numbers of rats entering central area **(G)** of rats in control, CUS (depression), CUS + REMSD, CUS + fluoxetine, and CUS + REMSD + fluoxetine groups were determined by Sucrose preference test and open field test. **(H, I)** Latency and space exploration time of rats in control, CUS (depression), REMSD, fluoxetine, and REMSD + fluoxetine groups were measured using Morris water task. ^***^P < 0.001, vs. control; ^#^*P* < 0.05, ^##^*P* < 0.01, ^###^*P* < 0.001, vs. CUS; ^∧∧∧^*P* < 0.001, vs. REMSD; ^&^*P* < 0.05, ^&&&^*P* < 0.001, vs. fluoxetine. REMSD, rapid eye movement sleep deprivation; CUS, chronic unpredictable stress.

To discover the possible effects of depression on rats, the rats were first exposed to CUS for the construction of depression, and their body weight was measured on days 1, 7, 14, 21, and 28 after model construction. As shown in Figure 1A, the rats in the CUS group showed a reduced body weight (*P* < 0.001), which suggested that depression decreased the rat body weight.

### Rapid Eye Movement Sleep Deprivation Combined With Fluoxetine Increased Body Weight and Prompted Behavior of Rats With Depression

After depression model construction by CUS and treatment with REMSD and fluoxetine, the body weight of rats was measured, and their capability of pleasure experience, exploration, and cognition was determined by the Sucrose preference test, the open field test, and Morris water task, respectively. Meanwhile, the locomotor activity of rats was monitored as well. In Figure 1B, it could be found that depression decreased the body weight of rats (Figure 1B, *P* < 0.001). However, the body weight of rats was increased after treatment with REMSD or fluoxetine alone, and it was further enhanced by co-treatment with REMSD and fluoxetine (Figure 1B, *P* < 0.05), suggesting that REMSD and fluoxetine treatment could promote the body weight of rats with depression.

The results from the Sucrose preference test and the open field test showed that after construction of the depression model, the consumed volume of Sucrose, the distance of horizontal movement, retention time for 5 min, and times entering the central region were decreased, whereas the immobility time was increased (Figures 1C–G, *P* < 0.001). However, REMSD or fluoxetine treatment increased the consumed volume of Sucrose and the distance of horizontal movement, retention time for 5 min, and times entering the central region but decreased the immobility time, and the effect was further enhanced by co-treatment with REMSD and fluoxetine (Figures 1C–G, *P* < 0.001). Meanwhile, from the results of the Morris water task, we observed a high latency but reduced times of space exploration after the model construction of depression (Figures 1H,I, *P* < 0.001). However, after REMSD or fluoxetine treatment, the latency was reduced, but the time of space exploration was increased, and the effects were further promoted by co-treatment of REMSD and fluoxetine (Figures 1H,I, *P* < 0.05).

### Rapid Eye Movement Sleep Deprivation Combined With Fluoxetine Alleviated Damage and Attenuated Apoptosis in the Hippocampi of Rats With Depression

**Figure 2:**
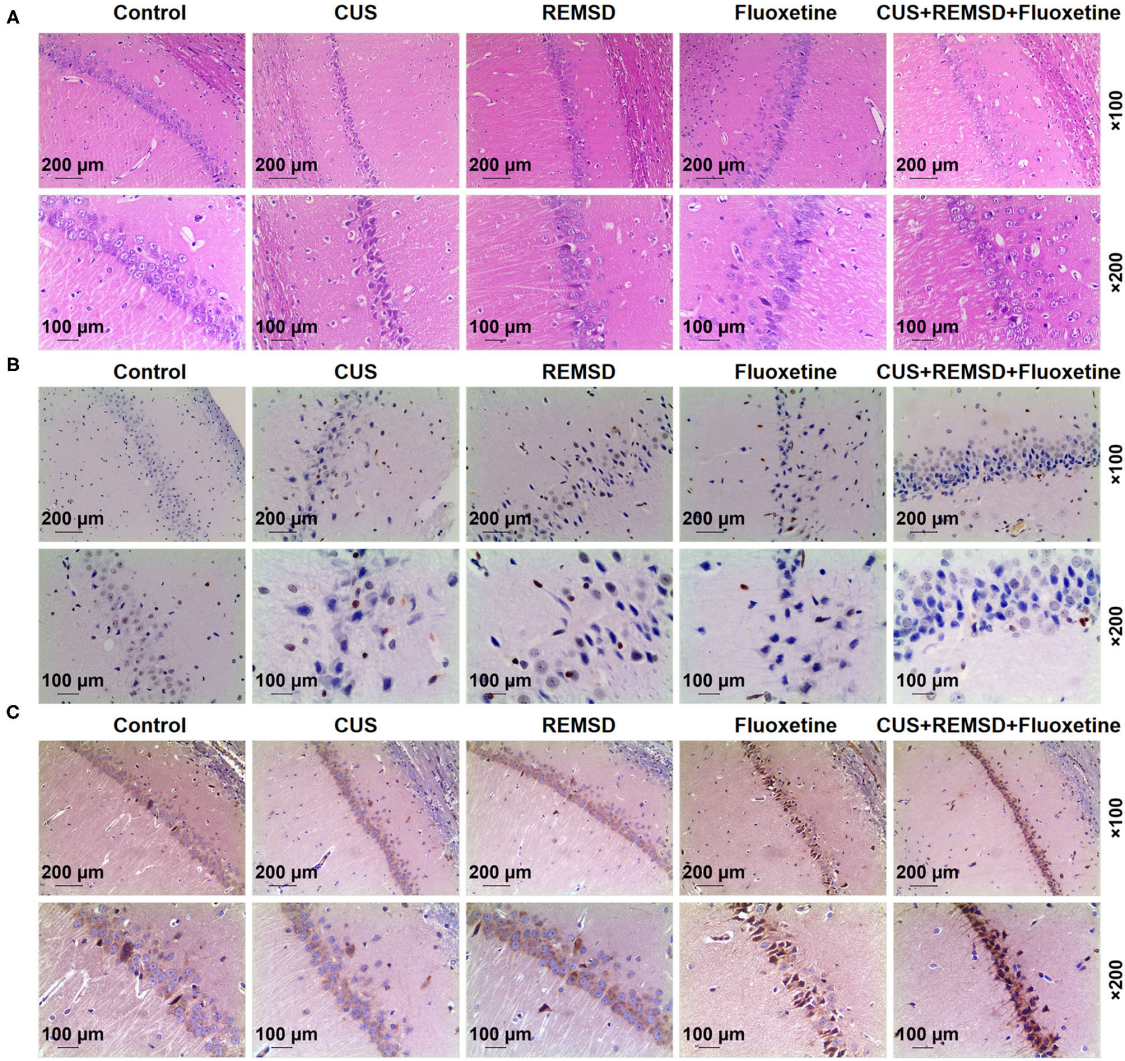
REMSD combined with fluoxetine alleviated damage, attenuated apoptosis, and promoted A1 adenosine receptor level in hippocampi of rats with depression. **(A)** Histology of hippocampi of rats in control, CUS (depression), REMSD, fluoxetine, and REMSD + fluoxetine groups was observed by H&E staining, under × 100 and × 200 magnifications. **(B)** Apoptosis in hippocampi of rats in control, CUS (depression), CUS + REMSD, CUS + fluoxetine, and CUS + REMSD + fluoxetine groups was determined using TUNEL assay, under × 100 and × 200 magnifications. **(C)** Immunohistochemistry analysis of A1 adenosine receptor in rats in control, CUS (depression), CUS + REMSD, CUS + fluoxetine, and CUS + REMSD + fluoxetine groups, under × 100 and × 200 magnifications. H&E, hematoxylin–eosin; TUNEL, terminal transferase-mediated biotin 2′-deoxyuridine, 5′-triphosphate nick end labeling; REMSD, rapid eye movement sleep deprivation; CUS, chronic unpredictable stress.

Then, all rats in the control, CUS, CUS + REMSD, CUS + fluoxetine, and CUS + REMSD + fluoxetine groups were killed, and the hippocampus tissues were subjected to H&E staining and TUNEL assay for detecting the damage and apoptosis in the hippocampi. In Figure 2A, the results from H&E staining showed that rats in the control group presented clear and normal hippocampi, whereas injured hippocampi were found in the CUS group (Figure 2A). It was also found that depression-induced hippocampus damage in rats was alleviated after REMSD and fluoxetine treatments and that the combined therapy of REMSD and fluoxetine caused a stronger effect on alleviating depression-induced hippocampus damage (Figure 2A). These results suggested that REMSD combined with fluoxetine could alleviate the damage in the hippocampi of rats with depression.

In Figure 2B, the results from the TUNEL assay demonstrated that the apoptosis in the hippocampi rats in the CUS group was more than that in the control group, whereas a lower apoptosis level in rat hippocampi was observed after REMSD and fluoxetine treatments (Figure 2B). In addition, the combined therapy of REMSD and fluoxetine could attenuate depression-induced apoptosis in rat hippocampi (Figure 2B). Thus, it could be summarized that REMSD combined with fluoxetine could attenuate the apoptosis in the hippocampi of rats with depression.

### Rapid Eye Movement Sleep Deprivation Combined With Fluoxetine Promoted A1 Adenosine Receptor Level in the Hippocampi of Rats With Depression

As shown in Figure 2C, the results from immunohistochemistry indicated that A1 adenosine receptor level in rats after depression model construction was decreased, whereas REMSD and fluoxetine treatments promoted A1 adenosine receptor level in rats (Figure 2C). In comparison, rats co-treated with REMSD and fluoxetine displayed a higher level of the A1 adenosine receptor (Figure 2C). These results demonstrated that REMSD combined with fluoxetine treatment could promote A1 adenosine receptor level in rats with depression.

### Rapid Eye Movement Sleep Deprivation Combined With Fluoxetine Had Regulatory Effects on the Expressions of the A1 Adenosine Receptor, Apoptosis- and Phosphoinositide 3-Kinase/ P38 Mitogen-Activated Protein Kinase Axis-Related Proteins, cFos, and Adenosine Deaminase RNA Specific 2 in the Hippocampi of Rats With Depression

**Figure 3:**
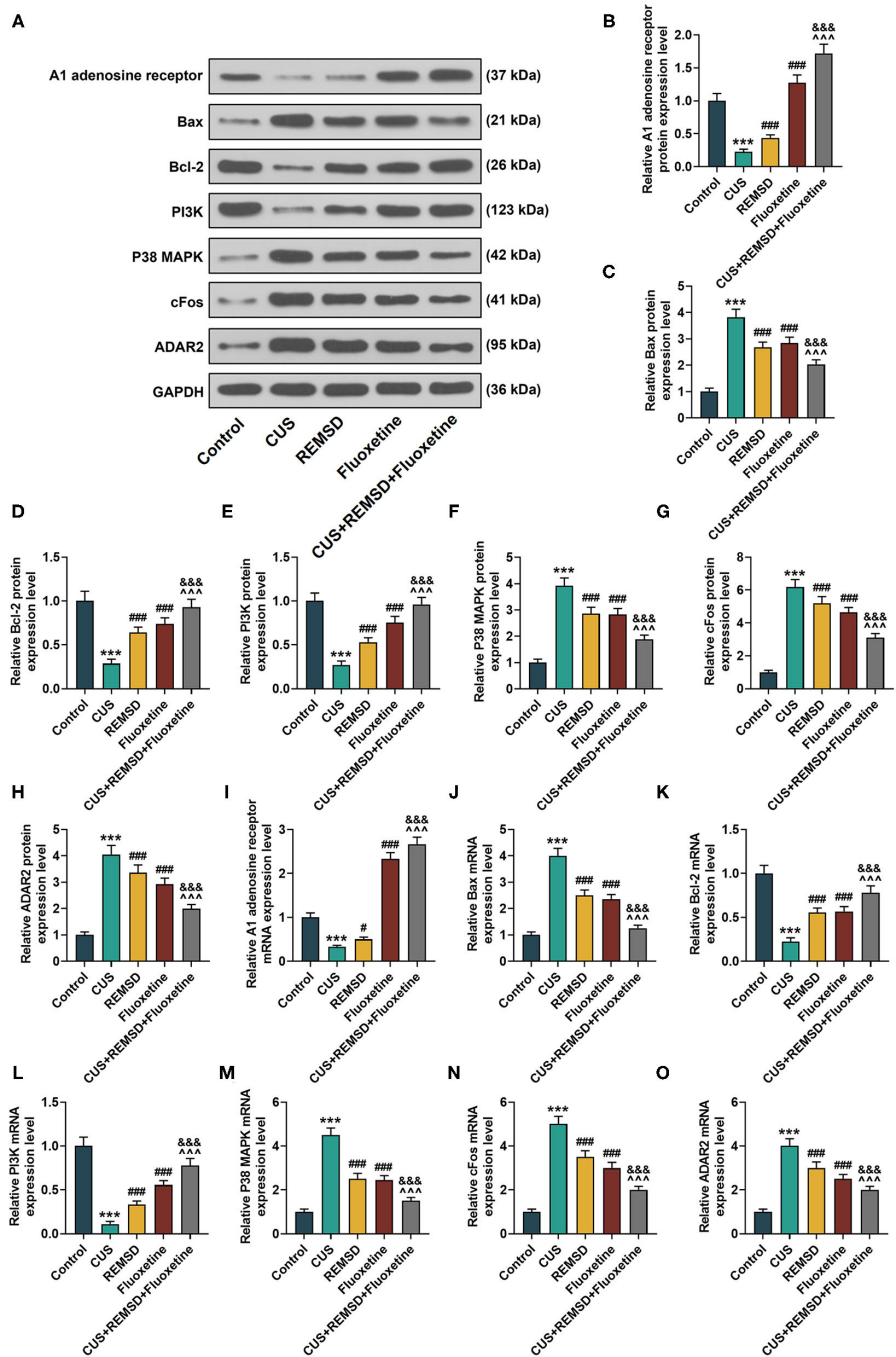
REMSD combined with fluoxetine had regulatory effects on expressions of A1 adenosine receptor, apoptosis- and PI3K/P38 MAPK axis-related proteins, cFos, and ADAR2 in hippocampi of rats with depression. **(A–H)** Relative protein/GAPDH expressions of A1 adenosine receptor, apoptosis- and PI3K/P38 MAPK axis-related proteins, cFos, and ADAR2 in rats of control, CUS (depression), CUS + REMSD, CUS + fluoxetine, and CUS + REMSD + fluoxetine groups were measured by Western blot. GAPDH was used as an internal control. **(I–O)** Relative mRNA expressions of A1 adenosine receptor, apoptosis- and PI3K/P38 MAPK axis-related proteins, cFos, and ADAR2 in rats of control, CUS (depression), CUS + REMSD, CUS + fluoxetine, and CUS + REMSD + fluoxetine groups were measured by qRT-PCR. GAPDH was used as an internal control. ^***^P < 0.001, vs. control; ^###^*P* < 0.001, vs. CUS; ^∧∧∧^*P* < 0.001, vs. REMSD; ^&&&^*P* < 0.001, vs. fluoxetine. Bax, Bcl-2-associated X protein; Bcl-2, B-cell lymphoma 2; PI3K, phosphoinositide 3-kinase; P38 MAPK, P38 mitogen-activated protein kinase; ADAR2, adenosine deaminase RNA specific 2; qRT-PCR, quantitative real-time polymerase chain reaction; REMSD, rapid eye movement sleep deprivation; CUS, chronic unpredictable stress.

To further confirm the effects of the combination therapy of REMSD and fluoxetine on rats with depression, the expressions of the A1 adenosine receptor, apoptosis- and PI3K/P38 MAPK axis-related proteins, cFos, and ADAR2 were measured by Western blot and qRT-PCR. In Figures 3A–O, the results from the Western blot and qRT-PCR showed that after the model of depression was constructed, the expressions of the A1 adenosine receptor, Bcl-2, and PI3K were downregulated, and those of Bax, P38 MAPK, cFos, and ADAR2 were upregulated (Figures 3A–O, *P* < 0.001). However, an opposite result was found after REMSD or fluoxetine treatment alone (Figures 3A–O, *P* < 0.05), and in comparison, the combined therapy of REMSD and fluoxetine further promoted the effects of REMSD and fluoxetine treatments on upregulating the expressions of the A1 adenosine receptor, Bcl-2, and PI3K and suppressing those of Bax, P38 MAPK, cFos, and ADAR2 in the hippocampi of rats with depression (Figures 3A–O, *P* < 0.001).

## Discussion

Cognitive impairment is a common, often persistent, symptom of MDD. The ultimate goal of treatment in depression is a full functional recovery, and evaluating patients with cognitive dysfunction for cognitive impairment and treatment selection should lead to improved functional outcomes (30). At present, approximately one-third of patients with depression who have received the treatment of either cognitive–behavioral therapy or antidepressant medication do not show any sign of improvement, and therefore, improvement of the treatment of depression is urgently required (31). The efficacy of SD and fluoxetine on the treatment of depression has been previously discussed (15). Nevertheless, REMSD and fluoxetine on the treatment of depression and the detailed molecular mechanism remained to be further uncovered. Our results provided evidence of the efficacy of the combined therapy of REMSD and fluoxetine on the treatment of depression, the interaction between the combined therapy and the A1 adenosine receptor, and the ameliorating effects of the combined therapy on depression-induced damage and apoptosis in the hippocampus.

Together with body weight dissatisfaction, body image, a complex concept which consists of several components, has prevailed in both adolescents and adults. Prior study has pointed out the association between body weight dissatisfaction and the onset of depression (32). Also, anhedonia has been recognized as the incapability to experience pleasure from rewarding or enjoyable activities and a core symptom of depression (33). Diagnosed anxiety disorders could predict later depressive disorders and *vise versa*, thereby showing the importance of identifying bidirectional risk factors for anxiety and depressive disorders (34). Meanwhile, cognition function impairment has been seen as one factor characterizing MDD, and circadian rhythm disruption is a core feature of mood and anxiety disorders (35, 36). A previous study demonstrated the effects of REMSD on hypocretin neurons in the hypothalamus of rats with depression (37). REMSD abrogated neurochemical and other depressive-like alterations (38). In our study, the effects of treatment with REMSD and fluoxetine alone or together on rats with depression were determined by the sucrose preference test, the open field test, and Morris water task; moreover, body weight measurement was also performed. We found that after the construction of the depression model, the rats showed a reduced body weight and restrained behavior, with decreased movement and increased latency. However, increased body weight and prompted behavior were found in rats after treatment with REMSD and fluoxetine alone, and the effect was further enhanced by the combination therapy of REMSD and fluoxetine, suggesting that the combined therapy of REMSD and fluoxetine could ameliorate rat depression. Nevertheless, the mechanism required further investigation.

Several signaling proteins could mediate neurotrophin signaling with different effects. PI3K protein belongs to the PI3K/Akt pathway and has a pro-survival effect on the hippocampus, and the activation of P38 MAPK is one of the central signaling transduction pathways that can trigger apoptosis (39, 40). It should be noted that the increase of apoptosis in the hippocampus is related to the depressive phenotype in mice with diabetes (41). Several proteins of the Bcl-2 family have been found to play important roles in the apoptosis in the hippocampus, for example, Bax has a pro-apoptosis role, and Bcl-2 exerts an anti-apoptosis function (42). In addition, in response to fear conditioning, principal hippocampal CA1 neurons activate several early genes, including cFos, which enables a rapid and lasting consolidation of contextual fear memory (43). ADAR2, a member of the ADAR family, is abundant in the hippocampus and important to the growth and development of the animal model (44). REMSD, as indicated previously, results in the activation of P38 MAPK to suppress the proliferation of adult hippocampal neural progenitor cells (45). In our study, we found that the combination therapy of REMSD and fluoxetine reversed the effects of depression on aggravating damage and promoting apoptosis in the hippocampus, upregulating the expressions of Bax, P38 MAPK, cFos, and ADAR2 expressions, and downregulating those of Bcl-2 and PI3K. This showed that the combination therapy could protect rat hippocampi against depression-induced damage and apoptosis, but the underlying molecular mechanism remained to be further elucidated.

Adenosine receptor subtypes (A1, A2, A2A, and A3) have been unveiled to have regulatory effects on various biological functions and play a role in diverse conditions, such as cerebral and cardiac ischemia, immune and inflammatory disorders, and cancer (19). It has been found that activation of the A2A adenosine receptor is related to the increase in depression-like symptoms, whereas upregulated A1 adenosine signaling is associated with the elicitation of antidepression effects (19). In addition, it was unveiled that SD could increase the density of the A1 adenosine receptor (46). In our study, we also found an increase in A1 adenosine receptor expression after treatment with REMSD and fluoxetine alone or together and that the combined therapy further promoted A1 adenosine receptor expression, showing that the combined therapy of REMSD and fluoxetine could protect rats against depression and rat hippocampi against depression-induced damage and apoptosis *via* promoting expression of A1 adenosine receptor.

We also focused on investigating possible mechanisms through which SD in combination with fluoxetine may provide better protection when compared with individual treatments. Fluoxetine is a serotonin reuptake blocker that suppresses REM sleep in mammals (47). The previous report showed that participants whose bedtime was delayed by 2 h in the night show the most significant improvement in negative affect and positivity ratio during the first 2 weeks of fluoxetine therapy (48). In this study, rats were deprived of sleep using the REMSD method. Thus, fluoxetine and REMSD may strengthen their impact on REM sleep deprivation.

This study design has limitations. Selective REMSD is not feasible in humans, which decreases the external validity and translational potential of our paper (but not its value from a physiological standpoint). Besides, accumulating evidence has indicated the role of REMSD as the cause and effect of mania (49). Thus, the antidepressant effect was not totally due to a reduction in depression but may be partially due to promotion on mania, which needed further exploration.

In conclusion, we provided evidence of the efficacy of the combined therapy of REMSD and fluoxetine on the treatment of depression and its interaction with the A1 adenosine receptor. Our present study also provided novel evidence of the protective effect of the combined therapy on the hippocampus against depression-induced damage and apoptosis. It is hoped that our current study can bring further insights into the pathogenesis of depression and provide potential methods for the prevention and treatment of depression in clinical practice in the future.

## Data Availability Statement

The original contributions presented in the study are included in the article/Supplementary Material, further inquiries can be directed to the corresponding authors.

## Ethics Statement

The animal study was reviewed and approved by the Committee of Experimental Animals of Zhejiang Chinese Medical University.

## Author Contributions

XJ and SW: substantial contributions to conception and design. PY, CZ, XH, JD, and ZT: data acquisition and data analysis and interpretation. XJ and SW: drafting the article or critically revising it for important intellectual content. XJ, SW, PY, CZ, XH, JD, and ZT: final approval of the version to be published and agreement to be accountable for all aspects of the work in ensuring that questions related to the accuracy or integrity of the work are appropriately investigated and resolved. All authors contributed to the article and approved the submitted version.

## Conflict of Interest

The authors declare that the research was conducted in the absence of any commercial or financial relationships that could be construed as a potential conflict of interest.
