# Supplementary figures and images for: Rapid Eye Movement Sleep Deprivation Combined With Fluoxetine Protects Against Depression-Induced Damage and Apoptosis in Rat Hippocampi via A1 Adenosine Receptor
Source: Front Psychiatry. 2021 Jul 16;12:599399. doi: 10.3389/fpsyt.2021.599399 (PMC8322534; doi:10.3389/fpsyt.2021.599399)

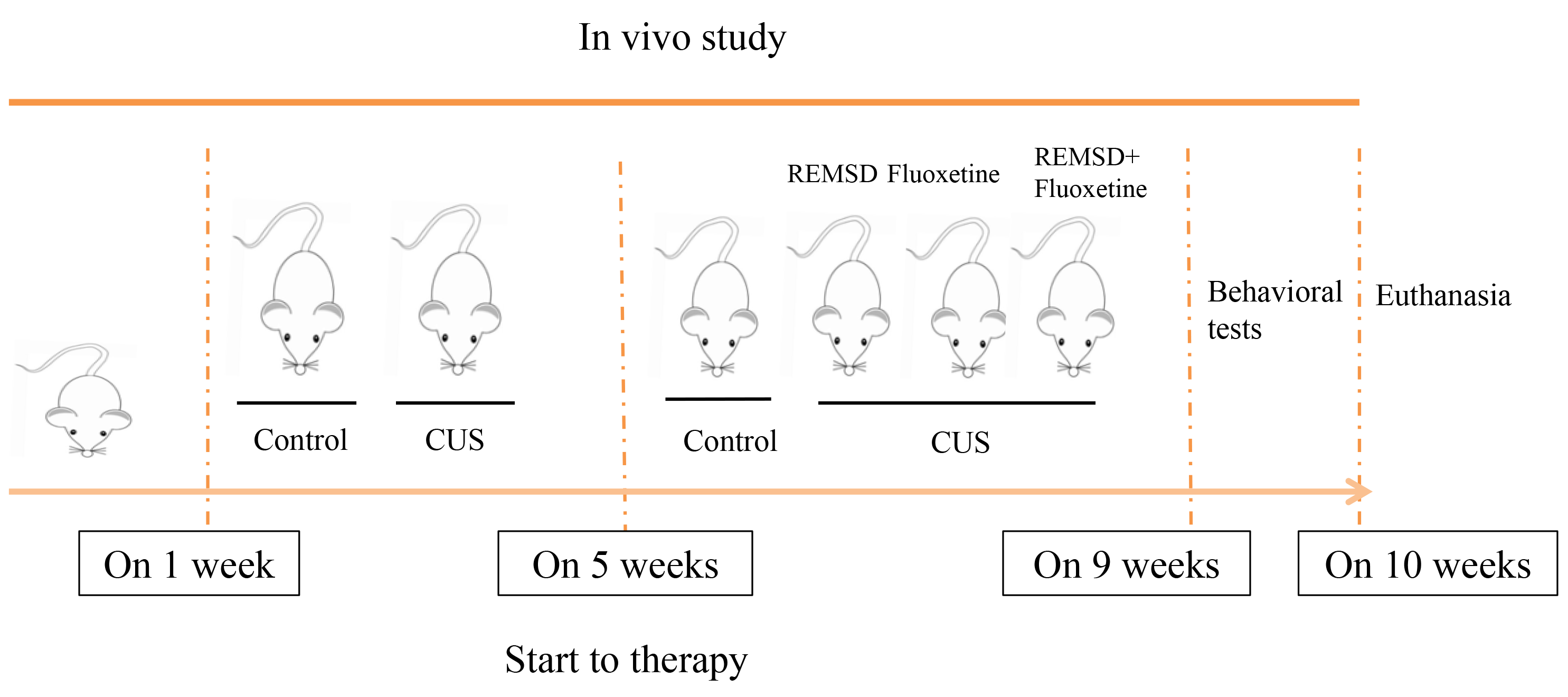

Supplement: Supplementary file 1 [file Image_1.TIF]
